# Some Practical Hints on Prostatectomy

**Published:** 1908-04

**Authors:** G. Frank Lydston

**Affiliations:** Chicago, Ill., Professor of the Surgical Diseases of the Genito-Urinary Organs in the State University of Illinois


For the Texas Medical Journal.

### Some Practical Hints on Prostatectomy.

BY G. FRANK LYDSTON, M. D., CHICAGO, ILL.,
Professor of the Surgical Diseases of the Genito-Urinary Organs in the State University of Illinois.

The technique of prostatectomy varies with the operator, but there are certain principles on which, it seems to me, the majority of operators should agree. There is, nevertheless, a wide variance of opinion on some very essential points. I present herewith, therefore, merely my own views as formed from a quarter of a century's observation and an experience of more than five hundred radical operations on the prostate.

*Indications for Operation.*—Every case of prostatic enlargement in which symptoms are present or the use of the catheter is required should be operated, with the following exceptions:

1. Men in full sexual vigor below 65 years of age in whom palliation is successful or symptoms are not severe and the urine is not infected. It may even be wise in exceptional cases to use the catheter, providing competent surgical assistance is always available In the exceptional cases in which the use of the catheter is permissible, the patient should plainly understand it is only a temporary expedient, and that the best he can hope for is a greater or less prolongation of his sexual life. Several operators have published operations in which the ejaculatory ducts are alleged to be systematically avoided, but, in my opinion, such claims are absurd. The illustrations appended to the descriptions of their alleged operations published in a prominent journal by several Eastern surgeons are "fake," pure and simple. No man living ever pried a prostate "baseball" fashion out of a median perineal incision and incised its capsule "under the eye" at will. Why every surgeon and anatomist in this country did not rise in wrathful protest against these "commercial" illustrations is a mystery. Much can be done to avoid injury to the ducts, it is true, but all the fraudulent pictures and descriptions on earth will not alter the anatomy of the prostate and perineum.

2. Inflammatory enlargement in relatively young subjects should not be "prostatectomized." I have seen a young man of 28 years of age submitted to prostatectomy for an ordinary chronic prostatitis. If I am ever called to the witness stand in a malpractice suit involving such an operation, I shall be tempted to treat the malefactor as he deserves. In very rare instances middle aged or old men with infected, chronically inflamed prostates may require prostatectomy. Old cases of prostatic abscess are best treated by prostatectomy.

3. Very old men who have already acquired the catheter habit, and who have used the catheter without trouble or detriment for some years should not be operated.

Malignant disease requires immediate operation, first, to radically remove the disease, or, second, to drain the bladder and give temporary relief. Primary cancer of the prostate is not rare; indeed, I have often met with it. I shall operate on one this very day. In general, a hard, nodular prostate of recent symptomatic development and rapid loss of flesh is indicative of carcinoma. Blood and "meat washings" in the urine or pain at night practically settle the diagnosis beyond peradventure of doubt. The older the subject, the better the prospect of a prolonged period of relief by operation for prostatic carcinoma. Malignant pathology marches slowly in the aged. I have just learned of the death of one of my cases two and one-half years after operation. He died of cancer beginning in the prostatic "space" and involving the gut, spleen and liver.

It is hardly necessary to state that apparently hopeless and moribund cases should not be submitted to prostatectomy. Palliative operations perhaps,—radical, never.

Prostatic enlargements without symptoms are not necessarily an indication for operation. Such cases may die of old age, yet never have the least annoyance from the prostates. As a rule, the operation should be done after the period of sexual life has elapsed, but in occasional cases a "waiting game" may be played. If, however, the patient is not situated so that he can be carefully watched and supervised, operation is imperative.

*Method of Operation.*—No given operation can be employed in all cases. The perineal route is best, but either the suprapubic or the combined may be required in certain cases. The median perineal incision seems to me to be best. The capsule of the prostate should be opened on the lateral urethral wall, as this affords the best chance of avoiding the ejaculatory ducts.

*Drainage.*—I believe in temporary drainage. The wound is deep and usually bathed in septic urine of greater or less virulence. The surgical principle is to my mind very plain. I use a double cannula perineal tube of aluminum, and if there is much hemorrhage—which is rare—I pack strips of iodoform gauze about the tube. The tube is removed on the third or fourth day. If it is allowed to remain longer it is likely to retard healing and promote fistula, besides it is best to get the patient up as quickly as possible.

*Complications.*—The principal complication to be dreaded is renal congestion or inflammation and uremia. This may be best avoided by hourly rectal doses of six ounces of faintly alkalinized warm water, *without sodium chloride.* A few drops of liquor potass. with twenty grains of potassium citrate serve the purpose.

The continuous flushing method—guttation—of the bowel is excellent.

*Sequelæ.*—Sterility, impotence, perineal fistula and incontinence of urine are the things which most concern the patient's mind. Sterility results whenever the ejaculatory ducts are severed. Impotency is not likely to result in patients who are sexually vigorous at the time of operation. There is danger of impotency, however, even under such circumstances. In older men of feeble power virility is quite likely to flicker out completely after prostatectomy. The temporary disuse of the sexual functions may have much to do with the subsequent failure of power. In an excellent proportion of my cases the patients have been both fertile and potent after operation. Fortunately, infertility is not often of consequence in elderly men. Sometimes, on the other hand, it is a very serious matter.

Permanent fistula is a condition which I have never met with after my prostatectomies. If the drainage tube be fixed well back toward the anus and removed promptly, fistula will not occur—save in certain very debilitated patients and in malignant cases. Here it is quite likely to follow.

Incontinence of urine should be rare. I practically never experience it in simple prostatectomies. In cases necessitating much mauling about or division of the internal sphincter vesicæ, it often occurs.

*Prognosis.*—Statistics of prostatectomy are notoriously unreliable, because of the wide variance in the ages and condition of the subjects operated. In subjects not too far advanced in years, with comparatively sound kidneys and bladder, the mortality is almost nil. As to cure, the patient who is successfully operated may be assured that his relief will be complete and permanent.

---

Pulmonary Emphysema.—The following combinations are recommended in cases of pulmonary emphysema:

℞ Potass. iodidi . . . . . . . . . . . . . . . . . . . . . . . . . . . . . . . ʒj

Tinct. hyoscyami . . . . . . . . . . . . . . . . . . . . . . . . . . . ʒiij

Elix. calisayæ, q. s. ad . . . . . . . . . . . . . . . . . . . . . . . ℥ij

One teaspoonful every three hours in water or milk. Or:

℞ Potass. iodidi

Tinct. belladonna, aa . . . . . . . . . . . . . . . . . . . . . . . ʒss

Spir. etheris comp . . . . . . . . . . . . . . . . . . . . . . . . . . ℥ss

Aq., q. s. ad . . . . . . . . . . . . . . . . . . . . . . . . . . . . . . . ℥iij

One tablespoonful three times daily in water.—*Jour. A. M. A.*

